# Knowledge, attitude, and practice of virtual consultation among outpatients at a teaching hospital in Malaysia

**DOI:** 10.1371/journal.pone.0289176

**Published:** 2023-12-20

**Authors:** Siaw Cheok Liew, Vinod Pallath, Yassir Rasali, Chan Choong Foong, Wei Han Hong, Maw Pin Tan

**Affiliations:** 1 Medical Education Research and Development Unit, Universiti Malaya, Kuala Lumpur, Malaysia; 2 Department of Clinical Competence, Perdana University-Royal College of Surgeons in Ireland, Kuala Lumpur, Malaysia; 3 College of Health Science, VinUniversity, Hanoi, Vietnam; 4 Department of Medicine, Faculty of Medicine, Universiti Malaya, Kuala Lumpur, Malaysia; UCSI University, MALAYSIA

## Abstract

**Introduction:**

During the coronavirus disease 2019 (COVID-19) pandemic, the use of virtual consultations has accelerated to ensure continued access to healthcare despite lockdowns and physical distancing measures. We aimed to determine the knowledge (awareness) of, attitude (acceptability) to, and practice (exposure) [KAP] of virtual consultations (VC), the demographic factors associated with poor KAP, and the correlation between the three KAP domains.

**Methods:**

A cross-sectional study, using a convenience sampling technique, was conducted from 13 September, 2021 to 28 November, 2021. We designed a 45-item VC KAP questionnaire. This was distributed to outpatient users attending cardiovascular, dermatology, geriatrics, haematology, endocrine, respiratory, gastroenterology, rheumatology, or neurology clinics at the University Malaya Medical Centre. It was completed during face-to-face, online, or telephone interviews. The data were analysed using SPSS version 24.0. Binary logistic regression was used to determine the demographic factors associated with KAP. Correlation between KAP domains was determined using Spearman’s rho (r). A p-value of <0.05 was considered statistically significant.

**Results:**

A total of 366 questionnaires were completed. Knowledge (awareness), attitude (acceptability), and practice (exposure) were considered good in 69.7%, 80.9%, and 24.6% of participants, respectively. There were no significant relationships between age, gender, ethnicity, and duration of hospital attendance (years) with knowledge (awareness), attitude (acceptability), and practice (exposure). A moderate positive correlation was seen between knowledge (awareness) and attitude (acceptability) (Attitude total [Atotal]) (r = 0.48, p<0.001), with no significant correlation between knowledge (awareness) and practice (exposure) (r = 0.04, p = 0.45), and attitude (acceptability) (Atotal) and practice (r = 0.01, p = 0.82).

**Conclusion:**

Overall, outpatient clinic users had good knowledge (awareness) of and were receptive towards VC but had poor practice (exposure). More opportunities for VC use in healthcare can increase exposure and subsequent utilisation. Interventions to increase the effectiveness of VC use should be explored in future studies.

## Introduction

The integration of technology in healthcare is increasingly evident through the utilisation of medical applications, online-based medical education, and telemedicine [1]. The move towards more digitalised healthcare delivery has the potential to promote healthcare equity and tackle health inequalities, especially through the provision of healthcare to remote rural areas and for people with physical disabilities [2]. This shift to technology-assisted medical care is aimed at optimising patient support at home. Virtual consultation (VC), a form of telemedicine, used for remote communication and consultation between healthcare professionals and patients [3], offers advantages to patients and the healthcare system. Indeed, VC is more cost-effective than traditional care of patients because of reduced travel time, including for the healthcare system, through reduced space utilisation [4].

The coronavirus disease 2019 (COVID-19) pandemic had necessitated several public health measures, including lockdowns and physical distancing [5]. With access to face-to-face consultations severely curtailed, clinicians and patients were compelled to resort to alternative solutions in order to mitigate the effects of the COVID-19 pandemic on non-COVID-related healthcare issues [6]. Globally, a huge reduction in outpatient visits occurred to accommodate increasing emergency visits [7]. This led to a rapid switch to VCs [8, 9].

Virtual consultation use has persisted despite the end of the pandemic and has been increasingly employed in outpatient medical consultations [10]. Although face-to-face consultations remain the gold standard in patient care, VC is employed widely to provide continuity of care [11]. However, patients remain unconvinced about its potential utility despite the benefits [12]. There are technical and regulatory challenges, compounded by healthcare professionals’ concerns regarding the potential for clinical harm, which have led to a reluctance in VC adoption [5]. However, despite the complexities of VC, research has documented that VC is safe and effective [13]. To increase VC uptake, it is important that public awareness and acceptability of VC use are optimised. Previous studies on VC acceptability have reported that patient preference for VC was dependent on the reason for the medical consultation [14]. For successful implementation, it is important to assess patients’ knowledge (awareness) of, attitudes (acceptability) to, and practice (exposure) of VC.

**Fig 1:**
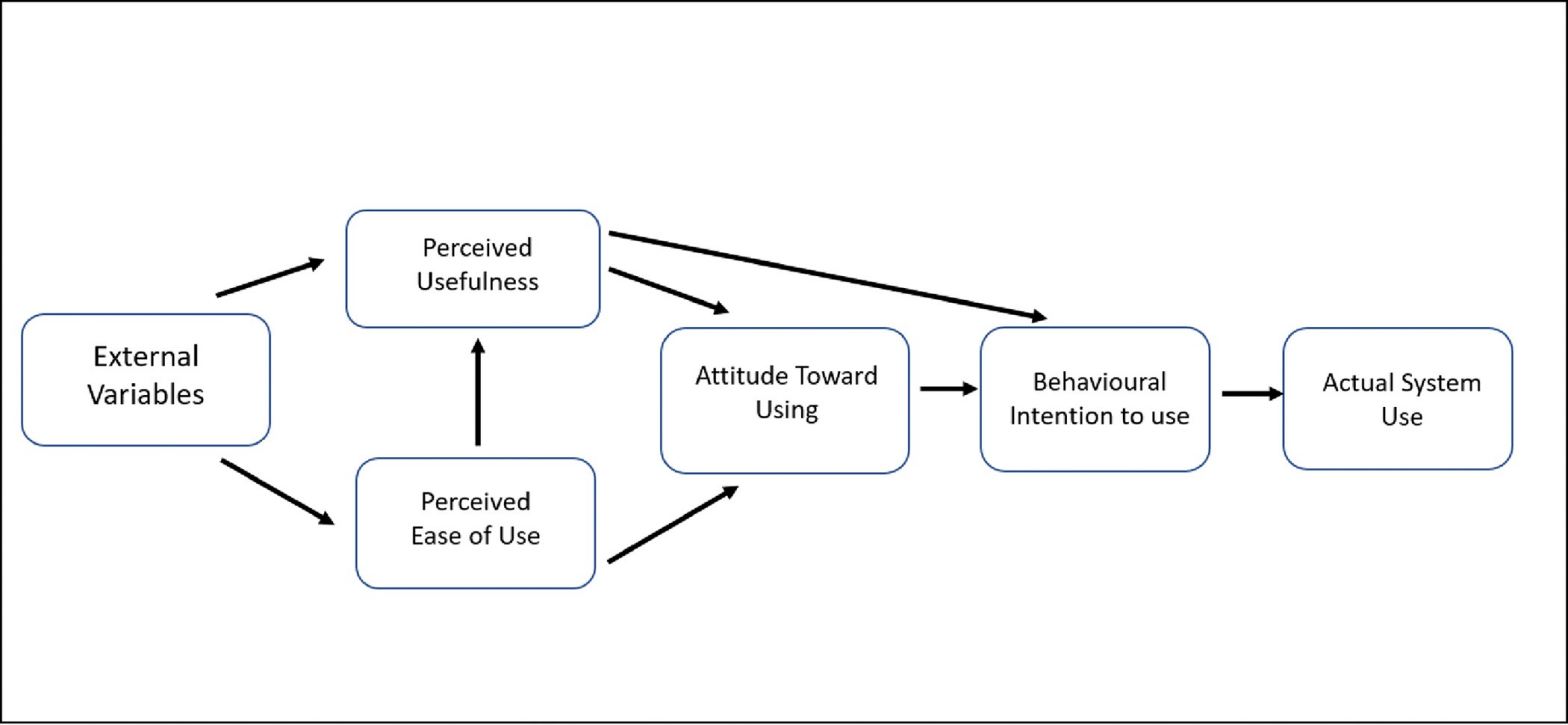
Technology Acceptance Model (TAM).

The Technology Acceptance Model (TAM), as depicted in Fig 1, denotes that users’ attitudes towards technology use are determined by the perceived usefulness and ease of use, which subsequently alters behavioural intentions and actual practices [9, 15]. The terminologies *attitude* for VC in this context, refers to patients’ acceptability, and *practice* refers to patients’ exposure and utilisation of VC. In essence, the TAM proposes that an effort to increase patient VC use requires reinforced awareness about its usefulness and ease of use.

Many studies have looked into the knowledge, attitudes, and practice (KAP) of healthcare professionals and medical students for telemedicine [16–18], but few studies have explored patients’ preparedness to embrace this method of healthcare delivery [19]. This study explores patients’ knowledge (awareness) of, attitude (acceptability) towards, and practice (exposure) of VC by determining their KAP score via a survey. The study also determines the association between demographic factors and KAP scores and correlations between KAP scores, in an attempt to elucidate factors that hinder or augment VC usage.

## Materials and methods

### Study design

A cross-sectional study was conducted among individuals accessing outpatient services at the University Malaya Medical Centre (UMMC), Kuala Lumpur, from 13 September, 2021, to 28 November, 2021. Participation in the study was voluntary. The inclusion criteria were patients aged 18 years and above that could read and write in English or Bahasa Malaysia (BM) (i.e., the national language).

### Sampling method

The estimated number of outpatient visitors attending the UMMC during the study period was 1.3 million. The sample size was calculated using Raosoft software ⓒ 2004 (Raosoft Inc, Seattle, USA) using a 95% confidence level and a margin of error of 5%. The estimated sample size was 385 responses. A convenience sampling method was applied. All patients who attended the outpatient department (cardiovascular, dermatology, geriatrics, haematology, endocrine, respiratory, gastroenterology, rheumatology, and neurology clinics) of UMMC and satisfied the inclusion criteria were invited to participate. In addition, some patients who regularly attended the outpatient clinics were telephoned and asked to participate. Willing participants were supplied with a written participant information sheet and provided written informed consent. Recruited patients completed a paper-based or online KAP questionnaire. This information was collated into REDCap version 9.9.0 (Vanderbilt University, Nashville, USA) which was used to produce an excel spreadsheet for data analysis.

### Knowledge, attitude, and practice questionnaire

This self-administered questionnaire collected patient demographic information, including age, gender, and number of years that the patient had been attending the hospital, in addition to information on VC knowledge, attitudes, and practice. The purpose of obtaining the demographic factors was to define the population in our setting that was obtained through convenience sampling. The KAP questionnaire was developed by the research team and validated by a panel of experts consisting of VC practitioners, medical practitioners, and health educationists. The questionnaire was initially developed in English and was subjected to the standard translation guidelines of “forward and backward” translation into BM. It was subsequently translated into BM by two bilingual experts, aiming to maintain the content meaning of the initial questionnaire. The translated questionnaire in BM was translated back into English by two additional bilingual experts, without any reference to the original English questionnaire. The translated questionnaire in BM was assessed for equivalency to the original and to the back-translated questionnaire by two research team members, S.C.L and A.Y.R. Some minor modifications were made. This observation, therefore, validated its adequacy in retaining the meaning and purpose of the translated questionnaire and substantiated the appropriateness of its use in this study.

In content validation, the panel assessed and identified the relevance and representativeness of the items included in the KAP questionnaire (See Tables 2 and 3). The item content validation index (I-CVI) value of >0.8 and an average-CVI (A-CVI) value of 0.93 was achieved. The questionnaire was subsequently distributed to 15 participants from the outpatient clinics and healthcare professionals to evaluate participants’ understanding of the questionnaire and to assess the relevance of the included items. The participants evaluated every questionnaire item and provided comments about the clarity, comprehensiveness, and relevance of each question in the open-ended comment section.

The difficulty index of the knowledge (awareness) questionnaire ranged from -3 to 3, except for item K3 (Table 2), and the discriminatory index was within a good range, except for items K2, K3, K5, and K15. All items were retained because they performed well in the item-goodness-of-fit model. The factor loadings for the attitude (acceptability) and practice (exposure) components were more than 0.40, except for item P1. The reliability of the questionnaire, using Cronbach’s alpha, was 0.75 for knowledge, 0.84 for attitude, and 0.88 for practice.

The VC KAP questionnaire had four questions pertaining to background demographics and forty-five items in the knowledge (awareness), attitude (acceptability), and practice (exposure) sections. The knowledge (awareness) section included 15 questions assessing general knowledge about VC availability, services provided via VC, and ethical considerations when using a VC. In the attitude (acceptability) section, 23 questions evaluated behavioural perceptions towards VC use in healthcare. This was divided into three sub-sections: AI, AII, and AIII (See Table 3) In the practice (exposure) section, 7 questions gathered the participant’s experience of VC in healthcare.

In the knowledge (awareness) section, incorrect or uncertain responses were assigned a score of 0, while correct responses were assigned a score of 1. The maximum total available score for the knowledge (awareness) section was 15. In the attitude (acceptability) and practice (exposure) sections, each was rated on a six-point Likert scale with a score of 1 indicating strongly disagree and a score of 6 indicating strongly agree. Negative statements were reverse-scored. The maximum total available scores for the attitude and practice sections were 138 and 42, respectively.

Participants’ KAP level was based on Bloom’s cut-off, which was termed “good” if a score between 80%-100% was achieved, “moderate” if the score was between 60%-79%, and “poor” if the score was less than 60% [20].

### Data analysis and statistics

Prior to data entry, all completed questionnaires were screened to ensure informed consent was appropriately signed and questionnaires were completely filled. Respondents with incomplete questionnaires were removed, as were those for when written informed consent was not available. Statistical analysis was conducted using the Statistical Package for Social Science (SPSS) version 20.0 (IBM^TM^, Armonk, USA). Continuous data are summarised using mean and standard deviation, with categorical data as frequencies and percentages. The association between the demographic status and KAP was evaluated using binary logistic regression. The outcomes were divided into two categories: “poor” and “good and moderate” KAP scores. The fundamental demographic factors of the population obtained were used only to understand their association with the KAP in our patient group. The correlations between the knowledge (awareness) of, attitude (acceptability) to and practice (exposure) of the participants towards VC were analysed using Spearman’s rho (r) correlation coefficient with 0.00–0.39, 0.40–0.69, 0.70–0.99, and 1.00 indicating weak, moderate, strong, and perfect correlation, respectively [21]. A p-value of <0.05 was considered statistically significant.

### Ethics approval

The study was approved by the Medical Research Ethics Committee of the UMMC (MRECID.NO: 2021130–9777). The research was carried out in accordance with the principles of the Declaration of Helsinki.

## Results

### Socio-demographic data

A total of 400 questionnaires were distributed, 371 respondents returned the questionnaires, of which 366 questionnaires were completely filled. The largest subgroup of participants was aged 36 to 45 years (23.8%), 193 (52.7%) were female, and had been attending the UMMC for less than 6 years. Respondent characteristics are summarised in Table 1.

**Table 1:** Demographic characteristics of participants (N = 366).

Variables		Frequency (n)	Percentage (%)
Age (years)	18–25	22	6.0
	26–35	58	15.8
	36–45	87	23.8
	46–55	62	16.9
	56–65	78	21.3
	66–75	51	13.9
	>75	8	2.2
Gender	Male	173	47.3
	Female	193	52.7
Ethnicity	Malay	179	48.9
	Chinese	112	30.6
	Indian	69	18.9
	Others	6	1.6
Length of time a UMMC patient (years)	<6	191	52.2
	6–10	102	27.9
	11–15	0	0.0
	16–20	24	6.6
	>20	49	13.4

Abbreviation: UMMC, University Malaya Medical Centre

### Participants’ knowledge of virtual consultation

A total of 254 (69.4%) participants obtained a total score of ≥9 in knowledge (awareness) about VC practice, which equated to an acceptable level. Out of a total score of 15, 138 (37.7%) participants had moderate knowledge (a score between 9–11), and 116 (31.7%) participants had good knowledge (a score between 12–15). Table 2 displays the aggregated scores for individual knowledge items. Although most of the participants (>79.2%) knew about the equipment needed and the ethical requirements of a VC encounter, some of them (ranging from 36.3%-70.8%) had a limited understanding of the scope of VC usage in healthcare. Binary logistic regression analyses revealed no statistically significant association between demographic factors and knowledge (awareness) in this patient group (Table 4).

**Table 2:** Knowledge of virtual consultations among visitors to the outpatient department (N = 366).

Questions		Frequency n (%)	
		Correct Answer	Wrong Answer
1	Can virtual consultation be used by healthcare professionals to provide healthcare services to patients remotely?	235 (64.2)	131 (35.8)
2	Does the use of virtual consultation require internet connectivity?	320 (87.4)	46 (12.6)
3	Does the use of virtual consultation require audio and video conferencing system?	290 (79.2)	76 (20.8)
4	Could virtual consultation be conducted using a mobile phone?	298 (81.4)	68 (18.6)
5	Could virtual consultation be used to obtain diagnosis and treatment?	133 (36.3)	233 (63.7)
6	Could virtual consultation be used to conduct screening and prevention of diseases?	136 (37.2)	230 (62.8)
7	Could virtual consultation be used to facilitate medical research?	240 (65.6)	126 (34.4)
8	Could virtual consultation be used to facilitate follow-up of patients?	259 (70.8)	107 (29.2)
9	Could virtual consultation be used for training of healthcare professionals?	223 (60.9)	143 (39.1)
10	Could virtual consultation be used for assessment of academic progress for healthcare professionals?	218 (59.6)	148 (40.4)
11	Could virtual consultation be used for peer-to-peer clinical communication among healthcare professionals?	278 (76.0)	88 (24.0)
12	Could virtual consultation be used to facilitate continuous medical education for healthcare professionals?	259 (70.8)	107 (29.2)
13	Should written consent be sought from patients, before virtual consultation can be conducted?	295 (80.6)	71 (19.4)

### Participants’ attitudes toward virtual consultation

Acceptable attitude (acceptability) towards VC was established in 296 (80.9%) respondents. A total of 154 (42.1%) participants had moderate, and 171 (46.7%) had good *“perceptions of the utility and acceptability of VC”* (AI Table 3). Altogether 325 (88.8%) participants, thought VC could reduce hospital visits, costs, and travel times, and hasten the specialist referral process.

**Table 3:** Attitude towards and practice of virtual consultation among visitors to the outpatient’s department (N = 366).

Questions		Favourable n(%)	Neutral n(%)	Unfavourable n(%)
**ATTITUDE**	***Perceptions of utility and acceptability of virtual consultation (AI)***			
**1**	I would consider virtual consultations as they would reduce hospital visits for patients.	**218 (59.6)**	**118 (32.2)**	**30 (8.2)**
**2**	I would consider virtual consultations as they would reduce cost for patients to see a doctor.	**220 (60.1)**	**118 (32.2)**	**28 (7.7)**
**3**	I would consider virtual consultations as they would reduce travelling time for patients.	**271 (74.0)**	**76 (20.8)**	**19 (5.2)**
**4**	I would consider virtual consultations as they would reduce time off work to see a doctor.	**244 (66.7)**	**102 (27.9)**	**20 (5.4)**
**5**	I would consider virtual consultations as they would increase patient’s engagement with the healthcare system.	**178 (48.6)**	**166 (45.4)**	**22 (6.0)**
**6**	I would consider virtual consultations as they would speed-up specialist referral and reduce appointment time.	**249 (68.0)**	**100 (27.4)**	**17 (4.6)**
**9**	I think virtual consultations are useful to manage my health problem.	**162 (44.3)**	**166 (45.3)**	**38 (10.4)**
**10**	I think virtual consultation should be practiced in the hospital that I visit.	**192 (52.5)**	**141 (38.5)**	**33 (9.0)**
**12**	I would like to receive training on the use of virtual consultations.	**174 (47.5)**	**153 (41.8)**	**39 (10.7)**
**13**	I would consider trying virtual consultations after attending training in virtual consultation use.	**185 (50.5)**	**149 (40.7)**	**32 (8.8)**
**14**	Virtual consultation training should be given to all healthcare practitioners, medical students and patients.	**230 (62.8)**	**112 (30.6)**	**24 (6.6)**
**15**	I would consider virtual consultations to receive healthcare in the future.	**224 (61.2)**	**120 (32.8)**	**22 (6.0)**
**16**	I would consider virtual consultation as an alternative way of receiving healthcare in the future.	**238 (65.1)**	**108 (29.5)**	**20 (5.4)**
	***Perceived challenges in virtual consultation (AII)***			
**17**	Virtual consultations threaten patient confidentiality and privacy.*	**120 (32.8)**	**186 (50.8)**	**60 (16.4)**
**18**	Virtual consultations limit effective patient-doctor communication.*	**155 (42.4)**	**167 (45.6)**	**44 (12.0)**
**19**	Virtual consultations increase a healthcare professional’s workload.*	**75 (20.5)**	**221 (60.4)**	**70 (19.1)**
**20**	Virtual consultations create new responsibilities for healthcare professionals.*	**122 (33.3)**	**180 (49.2)**	**64 (17.5)**
**21**	Learning to use virtual consultations requires a lot of effort.*	**133 (36.3)**	**176 (48.1)**	**57 (15.6)**
**22**	I think learning to use virtual consultation requires a lot of knowledge of IT gadgets.*	**158 (43.2)**	**145 (39.6)**	**63 (17.2)**
	***Factors enhancing acceptability of virtual consultation (AIII)***			
**7**	I would consider virtual consultations as it could reduce medical errors.	**80 (21.9)**	**209 (57.1)**	**77 (21.0)**
**8**	I think virtual consultations fit well with the way I like to receive healthcare.	**144 (39.3)**	**180 (49.2)**	**42 (11.5)**
**11**	I think I am comfortable with receiving healthcare through virtual consultations without any training.*	**89 (24.3)**	**181 (49.5)**	**96 (26.2)**
**23**	I would consider to practice virtual consultation because the reimbursement payment could be covered by the insurance company.	**111 (30.3)**	**202 (55.2)**	**53 (14.5)**
**PRACTICE**				
**1**	I have seen virtual consultation practiced in hospitals/clinics.	**71 (19.4)**	**119 (32.5)**	**176 (48.1)**
**2**	Virtual consultation is readily available and practiced in the hospital that I visit.	**44 (12.0)**	**161 (44.0)**	**161 (44.0)**
**3**	I have previously used virtual consultation to receive healthcare services.	**51 (14.0)**	**99 (27.0)**	**216 (59.0)**
**4**	I am currently using virtual consultation to receive healthcare services.	**41 (11.2)**	**118 (32.2)**	**207 (56.6)**
**5**	Virtual consultation technology training is implemented at the hospital/clinic that I visit.	**43 (11.7)**	**147 (40.2)**	**176 (48.1)**
**6**	I will be able to claim when receiving healthcare through virtual consultations via the insurance company/HR of my company.	**72 (19.7)**	**165 (45.1)**	**129 (35.2)**

* items to be reverse scored

Abbreviations: HR, human resource department

In the analysis of patients’ “*perceived challenges in VC”* (AII Table 3), a total of 270 (73.8%) participants showed poor attitude (acceptability). Participants did not perceive that VC may threaten patient confidentiality, or limit the patient-doctor relationship, nor did they think that VC use required much technical preparation. A total of 16 (4.4%) participants had a good perception and 192 (52.5%) had a moderate perception towards “*factors that enhance the acceptability”* of VC (AIII Table 3). These participants perceived that VC helped prevent medical errors, was a suitable platform for patients to receive healthcare, did not require much training, and that reimbursement would be made by the insurance company. Table 3 shows the breakdown of attitude (acceptability) scores of VC participants. Binary logistic regression showed no statistically significant demographic factors associated with attitude (acceptability) (Table 4).

**Table 4:** Binary logistic regression analysis of the association between demographic variables with poor knowledge (awareness), attitude (acceptability) and practice (exposure).

		Knowledge		Attitude		Practice	
		COR (95% CI)	p-value	COR (95% CI)	p-value	COR (95% CI)	p-value
**Gender**	Male						
	Female	1.43 (0.89–2.28)	0.137	1.05 (0.61–1.80)	0.871	1.58 (0.96–2.61)	0.073
**Age**	18–25		0.864		0.574		0.517
	26–35	2.55 (0.39–16.77)	0.332	1.93 (0.18–21.04)	0.591	1.64 (0.27–9.86)	0.589
	36–45	1.46 (0.25–8.59)	0.678	1.94 (0.20–18.56)	0.564	2.57 (0.49–13.59)	0.266
	46–55	1.93 (0.34–10.90)	0.455	1.00 (0.11–9.55)	0.999	2.22 (0.44–11.20)	0.333
	56–65	1.61 (0.28–9.33)	0.594	1.83 (0.19–17.29)	0.596	2.71 (0.52–14.08)	0.236
	66–75	1.34 (0.24–7.54)	0.738	1.41 (0.15–13.00)	0.762	2.36 (0.47–11.77)	0.296
	>75	1.68 (0.29–9.70)	0.565	2.49 (0.27–23.10)	0.423	4.82 (0.87–26.57)	0.071
**Ethnicity**	Malay		0.579		0.370		0.702
	Chinese	0.89 (0.12–5.06)	0.891	1.25 (0.14–11.46)	0.842	1.57 (0.27–9.18)	0.616
	Indian	0.64 (0.11–3.78)	0.626	0.88 (0.09–8.29)	0.911	1.44 (0.24–8.59)	0.691
	Others	0.99 (0.17–5.97)	0.997	1.75 (0.19–16.59)	0.626	2.15 (0.34–13.48)	0.415
**Duration as a patient in years**	<6		0.490		0.879		0.357
	6–10	0.73 (0.36–1.50)	0.392	1.08 (0.44–2.64)	0.870	0.53 (0.21–1.33)	0.175
	16–20	0.65 (0.29–1.41)	0.273	1.31 (0.52–3.33)	0.564	0.42 (0.16–1.09)	0.074
	>20	1.17 (0.44–3.07)	0.756	0.93 (0.27–3.24)	0.906	0.54 (0.16–1.78)	0.309

Abbreviation: COR, crude odds ratio; CI, confidence interval

### Participants’ practice of virtual consultation

Acceptable practice (exposure) of VC was established in 90 (24.6%) respondents. A total of 63 (17.2%) participants indicated moderate, and 27 (7.4%) indicated good practice (exposure) of VC. A small number of participants 131 (35.8%) had previously observed VC use in the hospital. About 116 (31.7%) participants had prior experience of using VC for healthcare, and 96 (26.2%) were currently utilising it. More than half of the participants, 235 (64.2%), reported that they would be comfortable utilising VC after attending training. Table 3 shows the breakdown of participants’ VC practice (exposure) scores. Binary logistic regression showed no statistically significant association between demographic factors with practice (exposure) (Table 4).

### Correlation between knowledge, attitude, and practice scores

There was a moderate correlation between knowledge (awareness) and attitude (acceptability) in general (Atotal) (r = 0.48, p<0.001). Specifically, a moderately positive correlation was seen between the knowledge (awareness) and attitude (acceptability) subsection AI—“perceptions of the utility and acceptability of VC” (r = 0.52, p<0.001) and a weak positive correlation between knowledge (awareness) and attitude (acceptability) subsection AIII—“factors that enhance the acceptability” (r = 0.24, p<0.001). There was no significant correlation between knowledge (awareness) and attitude (acceptability) subsection AII- “perceived challenges in VC” (r = 0.00, p = 0.95) and between knowledge (awareness) and practice (exposure) (r = 0.04, p = 0.45).

A weak negative correlation was found between attitude (acceptability) subsection AII and practice (exposure) (r = -0.16, p = 0.003) and a weak positive correlation was found between attitude (acceptability) subsection AIII and practice (exposure) (r = 0.26, p<0.001). There were no significant correlations between practice (exposure) and attitude (acceptability) (AI) (r = 0.05, p = 0.39) and between practice (exposure) and attitude (acceptability) (Atotal) (r = 0.01, p = 0.82).

## Discussion

Historically, VC research has been focussed on healthcare professionals’ awareness and acceptance [22–24], as well as the feasibility, accessibility, and suitability of this form of healthcare delivery [25]. This study provides insights into outpatient visitors’ knowledge (awareness) of, attitude (acceptability) towards, and practice (exposure) of VC, which have never previously been explored [26, 27]. Overall, the knowledge (awareness) of and attitude (acceptability) towards VC among outpatient visitors at this teaching hospital in Kuala Lumpur, the capital city of a higher-middle-income country in Southeast Asia, was high. In contrast, the practice (exposure) scores were low. This large divergence in knowledge (awareness) and attitude (acceptability) from practice (exposure) scores suggests that VC is an untapped resource.

The COVID-19 pandemic has revolutionised our lifestyles and working practices, with more being done online [27]. This enhanced engagement with virtual communication may explain the highly favourable attitude towards VC use and the observed high knowledge level. Further, a large amount of health information had been communicated virtually through social and conventional media during the pandemic. This is likely to have led to increased health literacy among patients, with an added appreciation for undisrupted access to healthcare, despite imposed restrictions on travel and physical contact [28].

Reed et al., [29] found that female patients favoured telemedicine consultations (either phone or video) over male patients. A similar finding was reported by Lam et al. [30] who discovered a higher prevalence of unreadiness for VC in male than female patients. The rationale for this gender bias remains unclear, with other studies demonstrating contradictory findings [31, 32]. Demographic factors, including gender, did not influence knowledge (awareness), attitude (acceptability), and practice (exposure) within this study, further refuting Lam et. al’s findings. It could be that women are more willing to try new things and to communicate virtually, either through telephone conversations or video calls [33]. Men, however, are often more attuned to technology, probably enhanced by cultural stereotyping, leading to more exposure to technology, even as children, within the home and formal education settings [34]. The two gender biases may, therefore, offset each other leading to no net gender difference in a predilection towards VC.

The adoption of VC within the Malaysian healthcare setting has not been as prevalent compared with many high-income countries [23]. The introduction of the Malaysian Telemedicine Act, 1997, has specified that the use of VC needs written, informed consent and physical examination [35], but a specific Malaysian Medical Council advisory has allowed for the management of patients with limited or non-use of physical examination in VC under special circumstances, such as the pandemic. Further, despite current literature revealing user satisfaction with virtual visits [36], our observation of poor VC practice (exposure) among outpatient visitors could have emanated from a lack of implementation, dissemination, availability, and training [37, 38]. The lack of correlation between knowledge (awareness) and attitude (acceptability) with practice (exposure) found in this study could, therefore, be explained by shortcomings in implementation. This contrasts with findings from previous studies, which show positive correlations between knowledge (awareness) and attitude (acceptability), knowledge (awareness) and practice (exposure), and attitude (acceptability) and practice (exposure) [18]).

**Fig 2:**
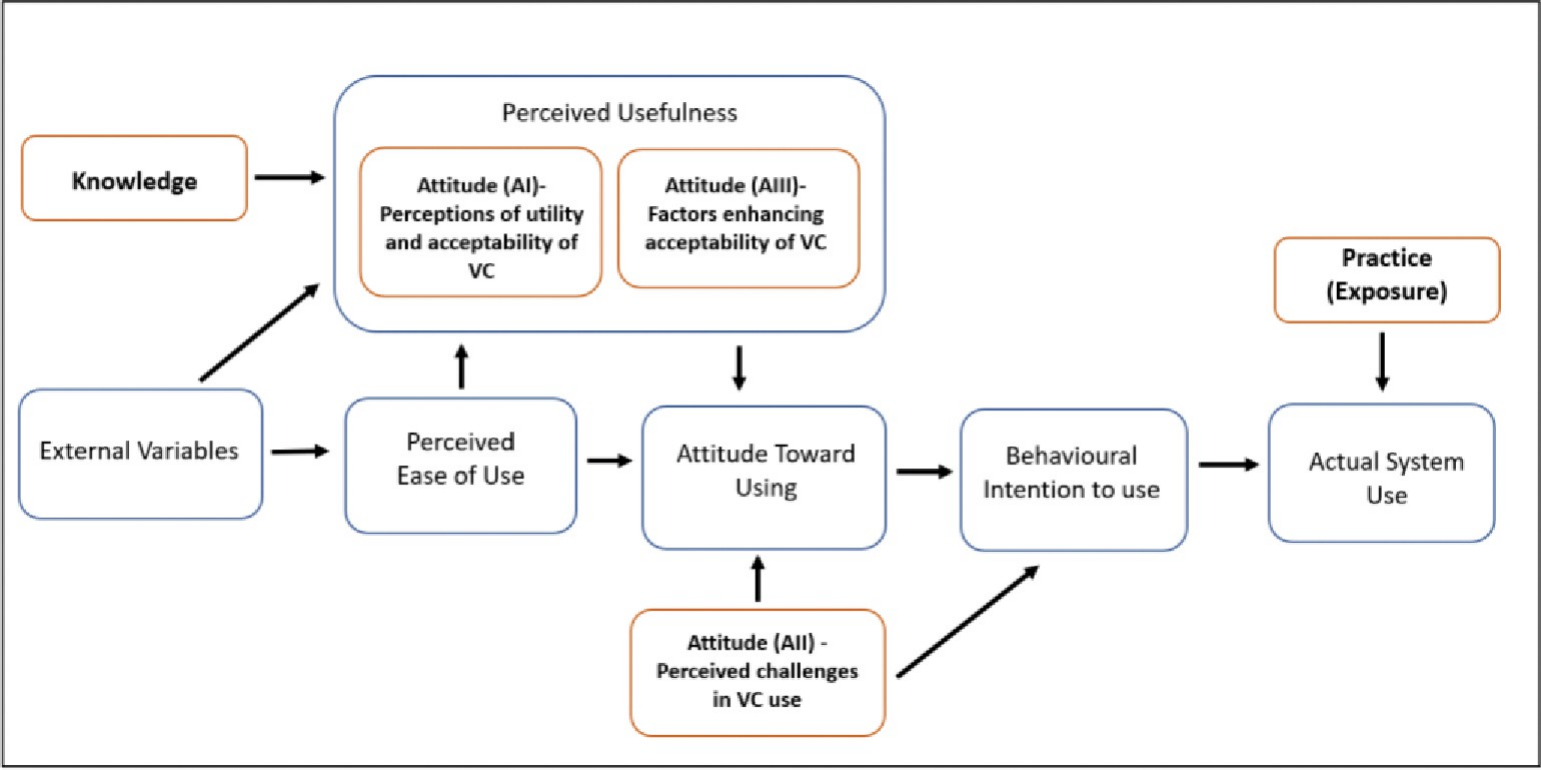
Modified TAM model for virtual consultation for patients.

Fig 2 shows the theoretical framework that evolved through this study, aligning with the TAM [15]. The acceptance of VC among outpatient department visitors is conceptually aligned with the perceived usefulness construct of the TAM. Perceived usefulness facilitates a positive attitude towards VC use, which in turn enhances behaviour, intention, and actual system use (practice). Actual system use is dependent upon an opportunity to use VC and exposure to it. The perceived challenges relating to confidentiality, privacy, and patient requirements for using this technology, if adequately addressed, should translate into effective VC use. This could be achieved through the provision of technical support to patients, to overcome any perceived challenges. Further, external factors, such as providing clarity on insurance reimbursement and charges for VC, are also important.

The original questionnaire was designed to capture the knowledge (awareness) of and attitude (acceptability) towards and practice (exposure) of VC among patients, medical students, and doctors. Questions 19 and 20 (See Table 3), concerning healthcare professionals in the “attitude” (acceptability) domain, had many patient responses that fell within the neutral category, suggesting that these were irrelevant to them. Hence, the above questions should be removed from the patient KAP questionnaire. Generally, the removal of redundant questions will lead to improvements in the psychometric properties of a questionnaire [39]. Our findings also indicate that Question 7 of the Attitude (acceptability) section could be reworded to *“the use of VC could cause medical errors”* and be reverse scored, to provide better meaning to the survey.

This study will serve as a reference point for future investigations into other factors that may contribute to patients’ VC KAP. The results of this study could also be used to review the TAM by elucidating factors that may augment or be considered as hindrances to the utilisation of VC, and regarding the changes/effort needed to boost patients’ acceptance of VC [15]. These efforts will possibly translate to effective VC utilisation and healthcare delivery.

This study was conducted in a single urban institution, which may have contributed to bias, as the results may not be representative of the whole population. Therefore, the responses from populations in rural areas and from underserved populations may not have been captured. A self-administered questionnaire may have contributed to selection bias as the lack of other local language translations may have limited participation. Future studies should attempt to address the discrepancies between knowledge (awareness) and attitude (acceptability) with practice (exposure) exposed within this study. In particular, the KAP could be re-evaluated after steps are taken to overcome existing barriers in VC implementation.

## Conclusion

Visitors to the outpatient department in this teaching hospital had good knowledge (awareness) of and attitude (acceptability) towards VC but had poor practice (exposure), which could be attributed to the lack of opportunities to use VC. The results show that patients are well-informed about VC and are ready to embrace it for healthcare delivery. Targeted interventions should be used to address factors that hinder VC utilisation and augment those that can boost its acceptability. Moving forward, there is a need to reflect on the experiences gained from VC use during the pandemic and to build on the currently available evidence, so that future educational programmes can effectively cater to the needs of the patients and healthcare professionals involved in VC utilisation and practice.

## Supporting information

S1 Data Raw data generated from the study. (XLSX)
